# Supplementary material for: Compartment specific responses to contractility in the small intestinal epithelium
Source: bioRxiv. 2023 Aug 7:2023.08.07.552224. Preprint. [Version 1] doi: 10.1101/2023.08.07.552224 (PMC10441304; doi:10.1101/2023.08.07.552224)
Supplement: Supplement 1 [file NIHPP2023.08.07.552224v1-supplement-1.pdf]

bioRxiv preprint doi: https://doi.org/10.1101/2023.08.07.552224; this version posted August 7, 2023. The copyright holder for this preprint (which was not certified by peer review) is the author/funder, who has granted bioRxiv a license to display the preprint in perpetuity. It is made available under aCC-BY 4.0 International license.

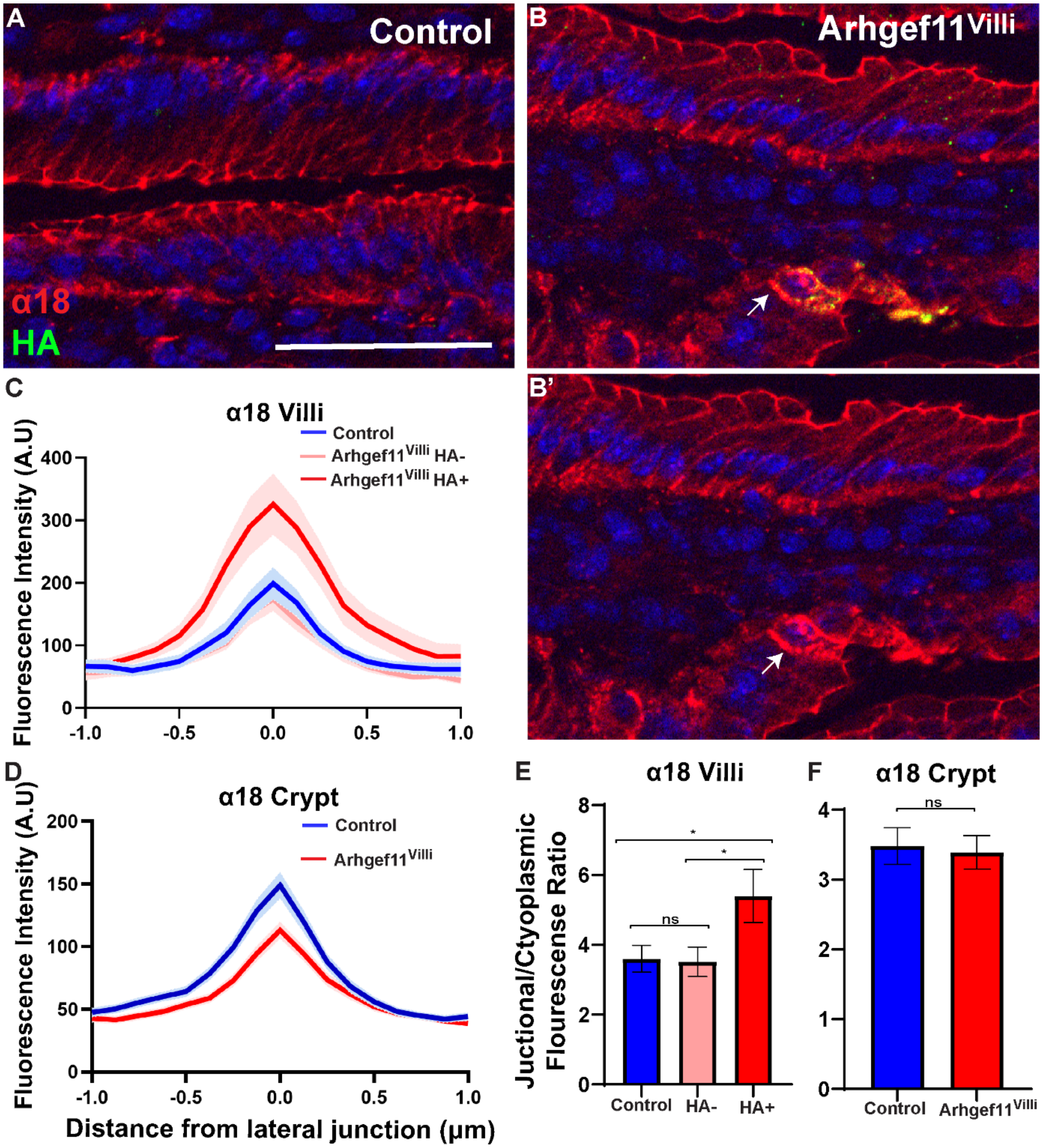

Supplemental Figure 1. Arhgef^{Villi} expression results in a local increase in adhesions junction tension.

(A and B) Immunofluorescence staining on small intestine sections of α-catenin α18 (red) and HA(green). Note that α18 staining is highest in HA+ cell in B'. Scale bar 50μm. (C and D) Quantification of lateral junction α18 intensity in (C) villar and (D) crypt cells. Data are mean ±SEM. (E and F) Average ratio of the junctional and cytoplasmic fluorescence values in (E) villi, p=0.0254, ordinary one-way ANOVA, n=24 cells for control and n=20 for HA- cells and n=18 HA+ cells for Arhgef^{Villi} and (F) crypts p=0.809, unpaired t-test, n=48 cells for control and n=47 for Arhgef^{Villi} from 3 mice per genotype.

bioRxiv preprint doi: https://doi.org/10.1101/2023.08.07.552224; this version posted August 7, 2023. The copyright holder for this preprint (which was not certified by peer review) is the author/funder, who has granted bioRxiv a license to display the preprint in perpetuity. It is made available under aCC-BY 4.0 International license.

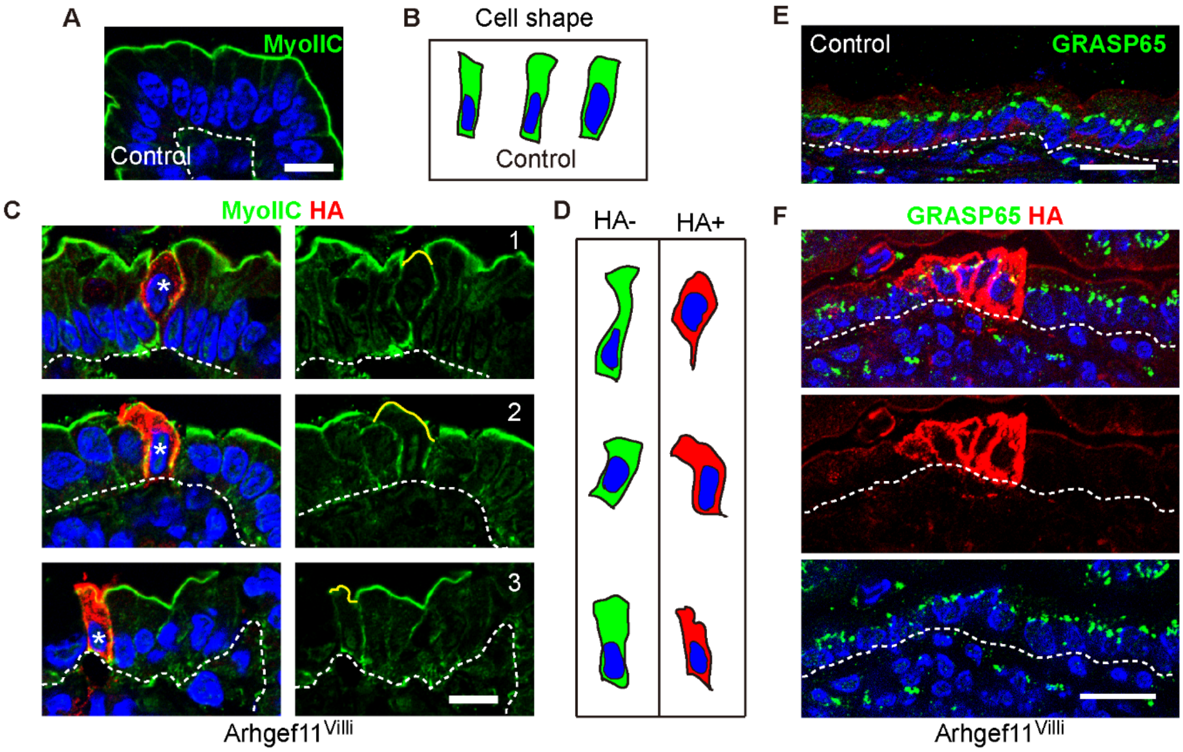

Supplemental Figure 2. Increasing junctional actin contractility deforms intestinal cell shape as well as the apical surface without affecting cell polarity.

(A and C) Immunofluorescence staining of MyoIIC (green) and HA (red) in control (A) and Arhgef^{Villi} (C) villar intestinal epithelia. Scale bars for A and C, 20 µm. (B and D) Traces of representative villar epithelial cell shapes in control (B) and Arhgef^{Villi} (D) HA-(green) and HA+(red) sections. (E and F) Immunofluorescence staining of GRASP65 (green) and HA (red) in control (E) and Arhgef^{Vill} (F) epithelial sections. Dotted lines mark the basement membrane. Scale bar for E and F, 40 µm.

bioRxiv preprint doi: https://doi.org/10.1101/2023.08.07.552224; this version posted August 7, 2023. The copyright holder for this preprint (which was not certified by peer review) is the author/funder, who has granted bioRxiv a license to display the preprint in perpetuity. It is made available under aCC-BY 4.0 International license.

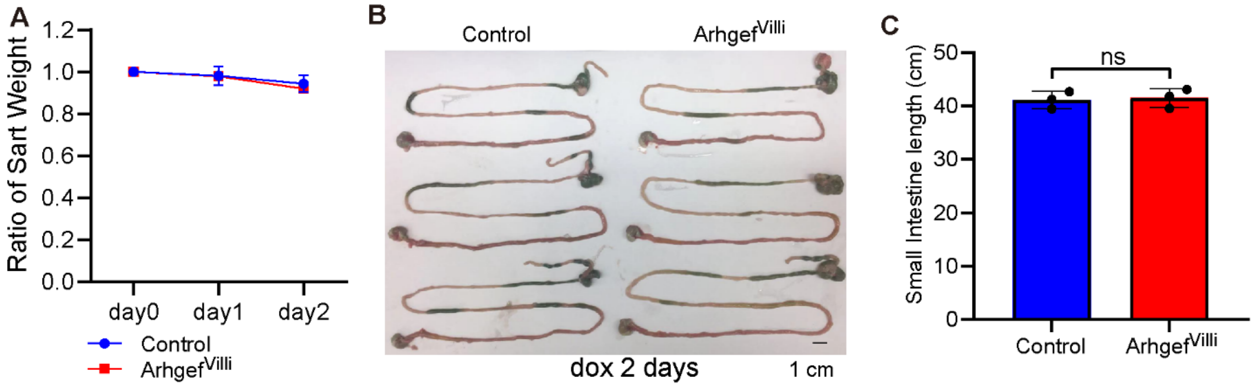

Supplemental Figure 3. Short term increased contractility in villar cells does not affect organismal health.

(A) Ratio of weight change after one day of doxycycline induction for control and Arhgef^{Villi}, n=3 animals for each genotype. (H) Control and Arhgef^{Villi} lower digestive tract images. Scale bar 1 cm. (I) Quantification of small intestine length of control and Arhgef^{Villi} animals in cm.

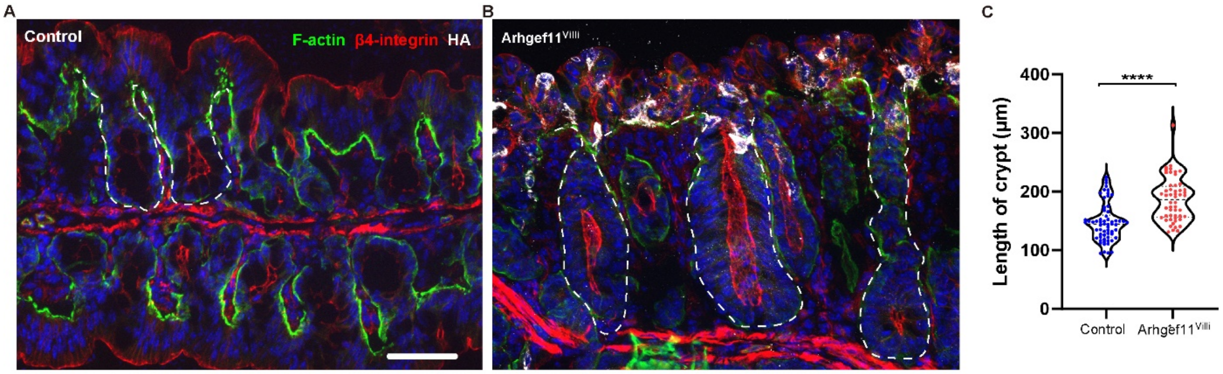

Supplemental Figure 4. Villin-rtTA driven cell contractility increases colon crypt length.

(A-B) Immunofluorescence images of (A) control and (B) Arhgef^{Villi} intestine sections stained with F-actin (green), β4-integrin (red), and HA (white). Dashed lines denote individual crypts. Scale bar 50µm. (C) Quantification of colon crypt length in microns. For control n=62 crypts from 3 animals. For Arhgef^{Villi}, n=52 crypts from 3 animals. p<0.0001, unpaired t-test.

bioRxiv preprint doi: https://doi.org/10.1101/2023.08.07.552224; this version posted August 7, 2023. The copyright holder for this preprint (which was not certified by peer review) is the author/funder, who has granted bioRxiv a license to display the preprint in perpetuity. It is made available under aCC-BY 4.0 International license.

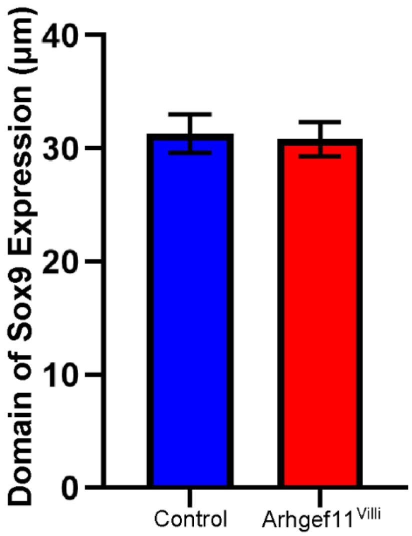

Supplemental Figure 5. Sox9 positive cell number remains unchanged upon increased villar cell contractility.

Average domain of the expression of Sox9 in crypt base stem cells. For control n= 62 crypts from 3 animals. For Arhgef^{Villi}, n=52 crypts from 3 animals. p=0.8316 ,unpaired t-test.

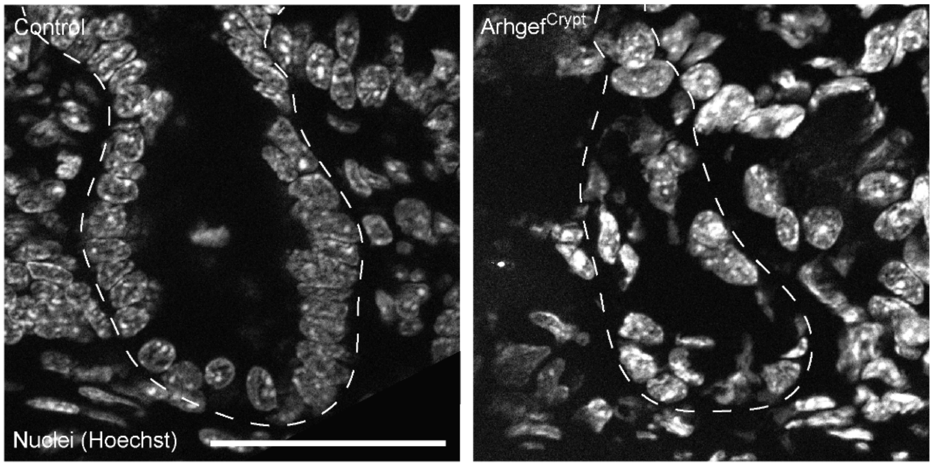

Supplemental Figure 6. Increased crypt cell contractility effects nuclear morphology.

Immunofluorescent images of control and Arhgef^{Crypt} crypt sections stained with Hoechst (white). Scale bar 50µm.
